# Factors associated with *de novo* aneuploidy across different preimplantation genetic testing cycles: a retrospective cohort study

**DOI:** 10.3389/fendo.2026.1805736

**Published:** 2026-05-28

**Authors:** Hongfang Liu, Hongxing Li, Xiaojuan Xu, Aiping Zhang, Bin Mao, Xiaoling Ma

**Affiliations:** 1 Reproductive Medicine Center, The First Hospital of Lanzhou University, Lanzhou, China; 2 Gansu Key Laboratory of Reproductive Medicine and Embryology, Lanzhou, China; 3 The First Clinical Medical College, Lanzhou University, Lanzhou, China

**Keywords:** aneuploidy, anti-Müllerian hormone (AMH), blastocyst morphology, preimplantation genetic testing, semen parameters

## Abstract

**Objective:**

Previous studies on factors influencing embryonic aneuploidy have produced inconsistent results. This study aimed to identify predictors of *de novo* aneuploidy in distinct patients undergoing preimplantation genetic testing for structural chromosome rearrangements (PGT-SR), aneuploidy (PGT-A) and monogenic disorders (PGT-M).

**Methods:**

We analyzed associations between maternal age, paternal age, anti-Müllerian hormone (AMH), maternal body mass index (BMI), controlled ovarian stimulation (COS) protocol, sperm parameters, blastocyst expansion degree, inner cell mass (ICM) grade, trophectoderm (TE) grade, and *de novo* aneuploidy using generalized linear mixed models (GLMM).

**Results:**

The incidence of *de novo* aneuploidy was 19.5% (PGT-SR), 32.1% (PGT-A), and 31.2% (PGT-M). Multivariate analysis showed that advanced maternal age and blastocyst expansion degree were significant predictors in the PGT-SR cohort. In the PGT-A cohort, significant associations were observed for maternal age, AMH, PGT indication, expansion degree, ICM grade, and TE grade. In the PGT-M cohort, TE grade was significantly associated with aneuploidy in women aged ≥ 30 years. No independent effects were found for semen parameters or stimulation protocols in any cohort. Whole-chromosome aneuploidies most frequently involved chromosomes 22, 16, and 21. There were no dominant chromosomes for segmental-chromosome aneuploidy. Notably, the aneuploidy rate remained consistently low (~20%) in women aged ≤ 30 years.

**Conclusion:**

In this study, advanced maternal age (≥35 years) emerged as a robust predictor of *de novo* aneuploidy. The expansion degree ex hibited an association with *de novo* aneuploidy across both the PGT-SR and PGT-A cohorts. The TE grade seemed to be correlated with *de novo* aneuploidy, especially in the PGT-A cohort, and the TE grade exhibited stronger predictive performance compared to the ICM grade. No independent predictive effect was detected for semen parameters or stimulation protocols. These findings support the implementation of individualized risk assessment and embryo selection in distinct PGT patient cohorts.

## Introduction

Embryonic chromosomal abnormalities serve as a critical biological mechanism underlying early pregnancy loss (1). In numerous cases, aneuploidy is hypothesized as the fundamental cause of embryonic arrest, implantation failure, and spontaneous pregnancy loss in both natural pregnancy and assisted reproductive technology (ART). Approximately 50-70% of all miscarriages can be ascribed to aneuploidy, and the exclusion of aneuploid embryos can significantly reduce the miscarriage rate after *in vitro* fertilization (IVF) (2). The selection of single euploid blastocysts for transfer is more likely to result in successful implantation and the establishment of a healthy pregnancy, thereby avoiding multiple pregnancies and associated complications (3).

Aneuploidy arises from meiotic irregularities, leading to an unequal distribution of chromosomes in gametes. This phenomenon is primarily associated with meiotic errors in the oocyte (4, 5). Aneuploidy increases exponentially with advancing maternal age (6). Another mechanism related to embryo aneuploidy is an error in post zygotic chromatid segregation during mitosis (7, 8). Consequently, factors associated with gamete meiosis or post-zygotic chromatid segregation during mitosis might also be related to embryonic aneuploidy.

Anti-Müllerian hormone (AMH), recognized as an ovarian reserve marker, indicates the magnitude of the primordial follicular pool (9, 10). A reduced level of AMH fundamentally signifies a reduction in the follicle count (11). There exists a dual tendency of a decline in both oocyte quality and quantity with advancing age (12). Simultaneously, in women aged over 35 years, the loss of adhesins and the deterioration of spindle monitoring function result in a notable increase in meiotic chromosome segregation errors (13). We hypothesize whether AMH can serve as an independent predictor of embryonic aneuploidy.

Research reports that the aneuploidy rates in donor egg cycles vary across different fertility clinics (14). The controlled ovarian stimulation (COS) process plays a significant role in ART by generating sufficient oocytes and euploid blastocysts. Hence, it is crucial to consider the potential impact of iatrogenic factors associated with ART such as COS protocol, which may contribute to the occurrence of embryonic chromosomal abnormalities.

Although the etiology of aneuploidy is predominantly ascribed to maternal factors, the role of the paternal factor and its magnitude remain inadequately comprehended (15, 16). The introduction of intracytoplasmic sperm injection (ICSI) revolutionized treatment, enabling males with sub-optimal semen quality, characterized by low sperm concentration, motility, and abnormal morphology, to reproduce. This also raises questions regarding the influence of these males on embryonic aneuploidy (17). Nevertheless, the sperm plays more of a role in fertilization than simply activating the oocyte and providing the paternal chromosomal contribution to the zygote. There is therefore concern about the potential incidence of chromosomal abnormalities when utilizing sperm with poor-quality parameters in ICSI treatments, especially as these may be associated with an increased rate of chromosomal aneuploidy in spermatozoa (18, 19).

Conventionally, embryo quality has been evaluated based on morphological characteristics. The blastocyst morphology grading system put forward by Gardner is the most widely acknowledged and extensively utilized for blastocyst selection in IVF (20). This system is based on the degree of blastocyst expansion and the morphology of the inner cell mass (ICM) and trophectoderm (TE) cells (21). Based on the Gardner morphological grading system, high-quality blastocysts were defined as those with a grade of ≥3BB (including grades 3, 4, 5, 6, AA, AB, BA, and BB), whereas poor-quality blastocysts were classified as grade C. Many studies have reported that blastocyst morphology parameters are significantly correlated with the implantation rate, clinical pregnancy rate, ongoing pregnancy rate, and live birth rate (22–24). The correlation between morphological assessment and embryonic aneuploidy that affects subsequent transfer outcomes remains poorly defined.

Preimplantation genetic testing (PGT), a crucial prenatal diagnostic approach, is employed to identify aneuploid embryos (25). This method can impede the transmission of pathogenic genetic mutations or unbalanced chromosomes to progeny, thereby enhancing the probability of a successful and healthy pregnancy. Consequently, PGT is extensively utilized in clinical settings.

Regardless of whether PGT-SR, PGT-A, or PGT-M is utilized, they all perform embryonic aneuploidy screening. This enables the exploration of the incidence of *de novo* aneuploidy in three PGT cohorts. A single-center retrospective study with a large sample was carried out to analyze the correlations of maternal age, paternal age, AMH levels, maternal BMI, PGT indication, COS protocol, sperm concentration, motility, morphology, DFI, blastocyst expansion degree, ICM grade, and TE grade with *de novo* aneuploidy at the blastocyst stage. This will facilitate our acquisition of a more profound comprehension of the patterns of embryo aneuploidy occurrence within diverse PGT populations and support genetic counseling for PGT patients.

## Materials and methods

### Patients and study design

The study involved 368 couples who underwent 400 PGT-SR cycles, 183 couples who underwent 200 PGT-A cycles, and 111 patients who underwent 119 PGT-M cycles from July 2020 to January 2025 at the center for reproductive medicine. A total of 662 couples and 719 cycles were performed. In the PGT-SR cohort, all the couples only one partner was a known carrier of a reciprocal translocation, Robertsonian translocation, or inversion, patients with compound chromosome abnormalities were excluded. Patients with advanced maternal age (AMA), recurrent pregnancy loss (RPL), and recurrent implantation failure (RIF) although they had normal chromosome karyotypes were assigned to the PGT-A cohort. Couples carrying monogenic diseases who sought fertility treatment were included in the PGT-M cohort. Patients with Y-chromosome microdeletion were excluded. The reference range for sperm DFI is defined by the WHO 6th laboratory manual: DFI ≤ 15% is normal, 15% < DFI < 30% is mild impairment, and DFI ≥ 30% is severe impairment. Since the proportion of participants with DFI ≥ 30% in this study is extremely small, dividing them into three groups would reduce statistical power. So, in this study, participants were divided into two groups based on DFI: DFI ≤ 15% and DFI > 15%. This study was performed in line with the principles of the Declaration of Helsinki. Approval was granted by the Ethics Committee of LZU No.1 Hospital (No. LDYYLL2025-2081). Informed patient consent was not required as the study was retrospective in nature and analyzed patient data anonymously.

### Controlled ovarian stimulation

Three stimulation protocols were used: a gonadotropin-releasing hormone (GnRH) agonist, a GnRH antagonist, and progestin-primed ovarian stimulation (PPOS). For the GnRH agonist protocol, during the 2–4 days of the menstrual cycle, a long-or short-acting GnRH agonist injection was given. After down-regulation criteria (based on hormone levels and antral follicle number) were met, gonadotropin (Gn) was administered with an initial dose of 100–300 U until the day of human chorionic gonadotropin (hCG) injection. When at least two dominant follicles had a diameter over 18 mm, 250 μg of recombinant hCG was injected for triggering, and oocytes were collected 34–36 hours later. In the GnRH antagonist protocol, Gn injection started on the second or third day of menstruation, with a starting dose of 100–300 U. A GnRH antagonist was injected between the 5–7 day of Gn-induced ovulation. When at least two follicles had a diameter over 18 mm, hCG was injected to promote oocyte maturation, and oocytes were retrieved 34–36 hours after the injection. The PPOS protocol involved simultaneously administering a short-acting GnRH agonist and Gn from the second to the third day of the menstrual cycle to stimulate the ovaries. When two or more follicles had a diameter of at least 18 mm, 250 μg of hCG was given to trigger oocyte maturation, and oocytes were retrieved 34–36 hours later.

### Embryo culture and trophectoderm biopsy

Mature oocytes only at the MII stage were fertilized by intracytoplasmic sperm injection (ICSI). Then, all the embryos were cultured in a sequential culture system (Vitrolife, Sweden). Embryos were biopsied on Day 5 or Day 6, according to the time of blastulation. Blastocysts were graded based on three distinct quality scores: the degree of expansion, the grade of the inner cell mass (ICM), and the grade of the trophectoderm (TE), using the Gardner morphological scoring system (20). Blastocysts graded above 3BC were utilized for subsequent biopsies. Five to ten trophectoderm cells were gently biopsied using a laser for zona drilling. Biopsied blastocysts were vitrified individually.

### Sample preparation and NGS procedure

The biopsy samples were rinsed using G-MOPS™ Plus medium and then put into 0.2 ml polymerase chain reaction (PCR) tubes with 2 μl of PBS. Next-generation sequencing (NGS) allows for the direct quantification of sequenced DNA fragments according to read numbers. Adhering to the Illumina NGS protocol, raw data were further handled through computational bioinformatics algorithms to map and align the short sequence reads to a linear human reference genome sequence. A small number of cases where DNA amplification did not succeed were excluded from the study. The minimum detection range equaled 4 Mb. An embryo was regarded as “abnormal” when the result differed from the reference baseline. Embryos with < 30% aneuploid cells were considered euploid, those with 30-70% aneuploidy were classified as mosaic and those with > 70% were considered aneuploid. This classification is explicitly recommended by the PGT-A Consortium consensus statement and is routinely adopted in major clinical studies to distinguish true mosaicism from euploid/aneuploid calls and technical noise (26). Based on chromosomal outcomes, embryos were classified into three categories: euploidy, aneuploidy, and mosaicism. Aneuploidy included whole-chromosomal aneuploidy and segmental-chromosomal aneuploidy. Among these, aneuploidy encompassed pure aneuploidy and aneuploidy combined with mosaicism. Only embryos with sole mosaicism were categorized as mosaic. Notably, within the PGT-SR cohort, aneuploidy encompasses both genetic aneuploidy and *de novo* aneuploidy. When aneuploidy solely originated from parental genetic abnormalities, it was categorized as genetic aneuploidy; otherwise, it was classified as *de novo* aneuploidy. For example, if parents were carriers of reciprocal translocation and aneuploidy only resulted from the 18 meiotic segregation gametes of reciprocal translocations, it was categorized as genetic aneuploidy; otherwise, it was regarded as *de novo* aneuploidy. In the PGT-SR cohort, parental chromosomal abnormalities increased the risk of embryonic aneuploidy (primarily referring to genetic aneuploidy). Moreover, the incidence of genetic aneuploidy adhered to a mechanism that was completely different from *de novo* aneuploidy. To facilitate parallel comparison among the three PGT cohorts, we discussed the influencing factors of *de novo* aneuploidy.

### Statistical analysis

Statistical analyses and data visualization were conducted using SPSS 27.0 (IBM SPSS Inc) and R statistical software, version 4.5.2. Categorical variables were presented as absolute values and percentages. Categorical variables were compared using the Pearson χ^2^ test or Fisher exact test. Continuous variables were reported as mean ± standard deviation (SD) or median (Q1, Q3). Statistical significance was evaluated using the ANOVA or Kruskal-Wallis test to compare continuous variables. A generalized linear mixed model (GLMM) with a binomial distribution and logit link function was employed to account for the non-independence of blastocysts derived from the same cycle. Cycle ID were included as random effects, while exposure variables and covariates were treated as fixed effects. Two multivariate models were constructed to identify independent predictors of *de novo* aneuploidy. Clinically guided full model: All variables with potential clinical relevance to the outcome were included, based on univariate analysis outcome, previous literature, and clinical plausibility. AIC-selected parsimonious model: A backward stepwise variable selection procedure was performed to minimize the Akaike Information Criterion (AIC), removing variables that did not contribute to model fit. Both models were compared to identify robust independent predictors. The data were reported as odds ratios (ORs) with 95% confidence intervals (95% CIs). All statistical tests were two-tailed. To control the false discovery rate (FDR) introduced by multiple comparisons in univariate and multivariate analyses, raw *P* values were adjusted using the Benjamini-Hochberg procedure. The FDR-adjusted *P* values were reported, and a significance level of adjusted *P* < 0.05 was applied for all analyses.

## Results

### General characteristics of the study subjects

**Figure 1:**
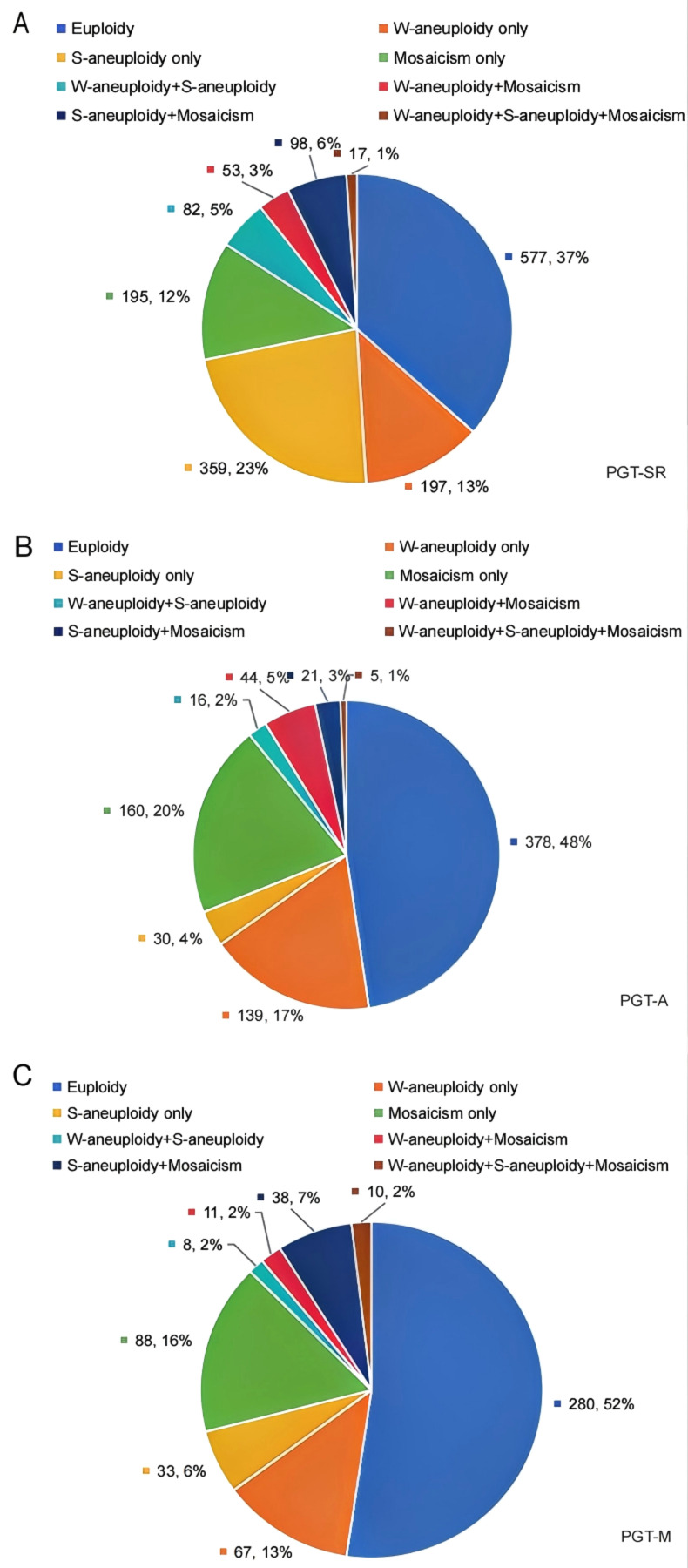
Summary of NGS-based PGT results in all biopsied blastocysts **(A)** PGT-SR cohort. **(B)** PGT-A cohort. **(C)** PGT-M cohort. W-aneuploidy, whole chromosomal aneuploidy; S-aneuploidy, segmental chromosomal aneuploidy. .

**Figure 2:**
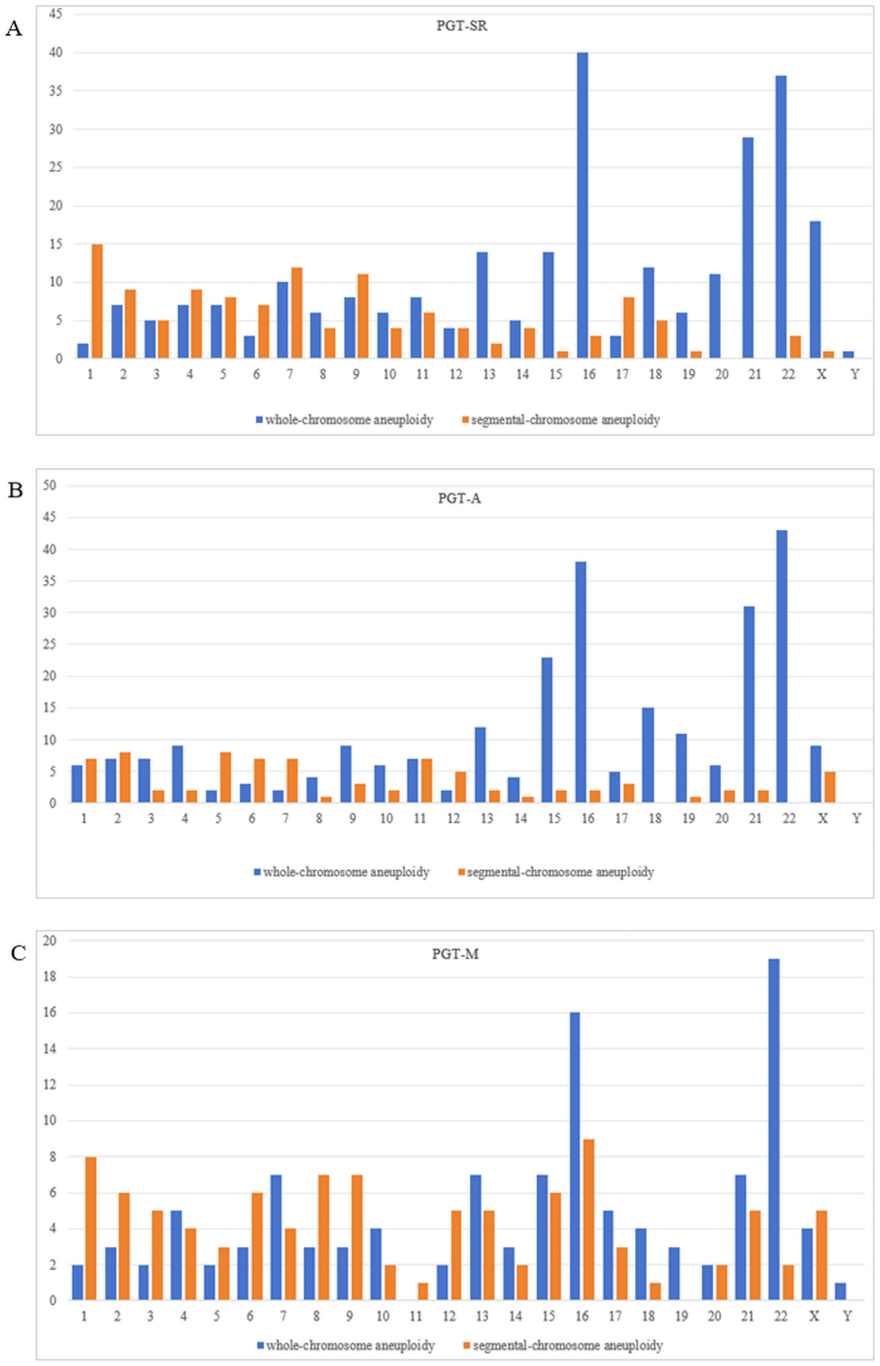
Distribution of *de novo* aneuploidy and types of *de novo* aneuploidy across the chromosomes in the three PGT cohorts. **(A)** PGT-SR cohort. **(B)** PGT-A cohort. **(C)** PGT-M cohort.

**Figure 3:**
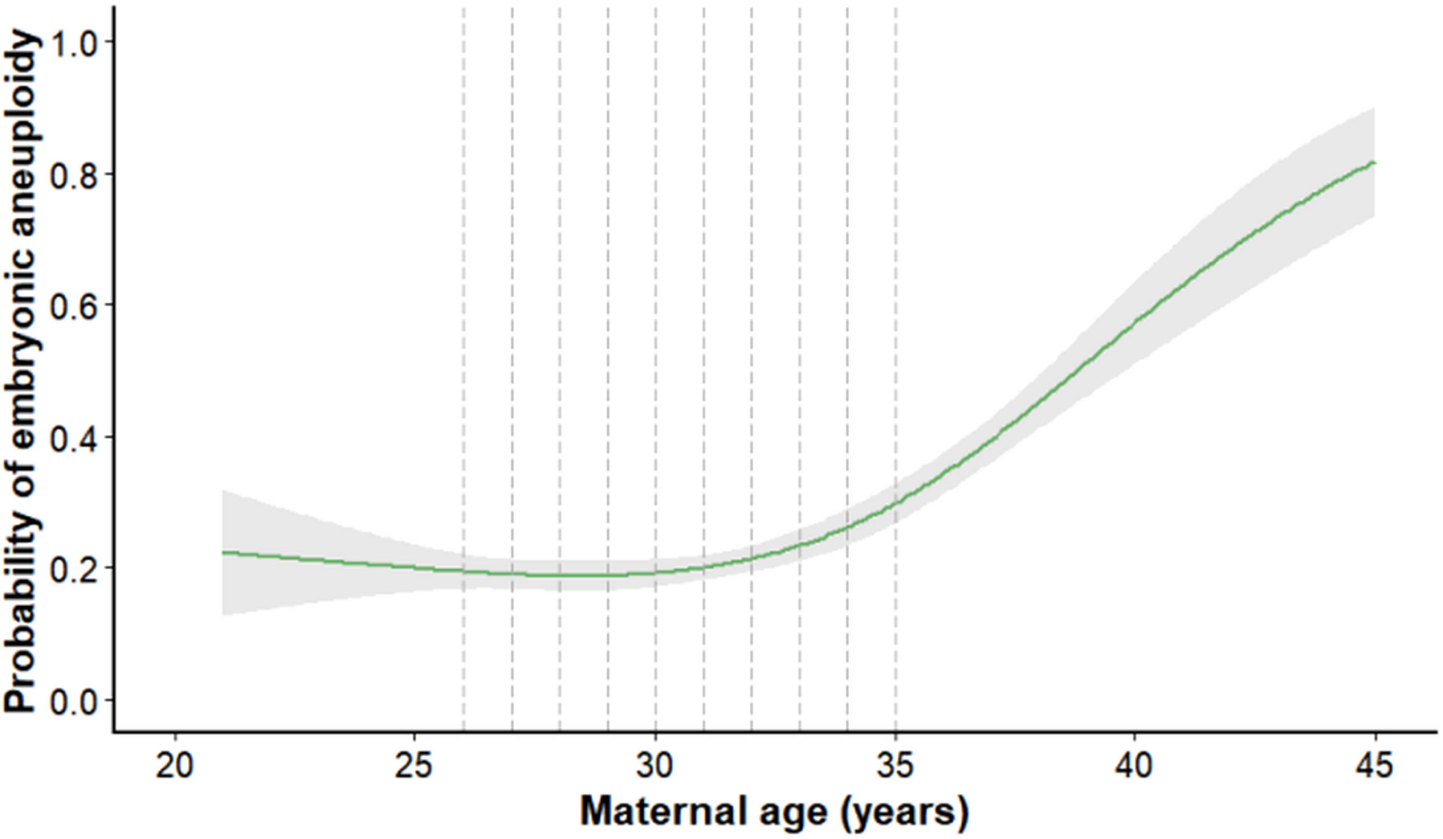
Association between maternal age and *de novo* aneuploidy probability.

The general demographic parameters and clinical characteristics were presented in **Table 1**. Twenty-five blastocysts were excluded from the analysis because they failed to meet the requirements for blastocyst biopsy or experienced DNA amplification failure during the whole-genome amplification (WGA) procedure. A total of 719 cycles from 662 couples, involving 2906 blastocysts, were included in this study. In the PGT-SR cohort, 36.6% of the blastocysts were euploid, 51.1% were aneuploid (including genetic aneuploidy and *de novo* aneuploidy), and 12.3% only carried mosaicism. In the PGT-A cohort, the proportions were 47.7% euploid, 32.1% aneuploid, and 20.2% mosaic embryos, respectively. In the PGT-M cohort, the corresponding data were 52.3% euploid, 31.2% aneuploid, and 16.5% mosaic. An overview of the blastocyst euploidy status was presented in **Figure 1**. The distribution of *de novo* aneuploidies among three PGT cohorts was presented in **Figure 2**. Whole-chromosome aneuploidy most frequently occurred in chromosomes 22, 16, and 21. However, there were no dominant chromosomes for segmental-chromosome aneuploidy. The association between maternal age and blastocyst aneuploidy rates was shown in **Figure 3**. When women were in the age range of 26 to 30, the aneuploidy rate of blastocysts remained relatively low. Around 35 years, the probability began to increase sharply (slope steepens), which aligned with the clinical definition of “advanced maternal age”.

**Table 1:** Patient characteristics and corresponding PGT data.

Variables	PGT-SR	PGT-A	PGT-M	*P* value	Total
Number of couples, n	368	183	111	–	662
Number of cycles, n	400	200	119	–	719
Maternal age, years	30.0 ± 3.5	33.9 ± 4.2	31.3 ± 4.2	<0.001	31.3 ± 4.2
Paternal age, years	31.6± 4.5	34.8 ± 4.7	34.2 ± 4.7	<0.001	32.6 ± 4.7
Maternal BMI, kg/m^2^	22.1 ± 2.7	22.3 ± 2.9	22.1 ± 2.4	0.424	22.1 ± 2.7
AMH, ng/ml	2.9 (1.7, 4.9)	3.2 (1.7, 5.6)	3.3 (2.1, 6.3)	0.005	3.05 (1.74, 5.17)
Retrieved oocytes, n	5928	3099	1955		10982
Mature oocytes (MII), n (%)	5092 (85.9%)	2659 (85.8%)	1685 (86.2%)	0.925	9436 (85.9%)
Cleavage embryos, n (%)	3625 (71.2%)	1848 (69.5%)	1178 (69.9%)	0.256	6651 (70.5%)
Blastocysts obtained, n (%)	1590 (43.9%)	803 (43.5%)	538 (45.7%)	0.455	2931 (44.1%)
Blastocysts with genetic results, n (%)	1578 (99.2%)	793 (98.9%)	535 (99.4%)	0.345	2906 (99.2%)
High-quality blastocyst, n (%)	843 (53.4%)	420 (53.0%)	270 (50.5%)	0.492	1533 (52.8%)
Cycles with at least one euploidy embryo (%)	72.8%(291/400)	81.5%(163/200)	84.9% (101/119)	0.005	77.2% (555/719)
Euploid blastocysts, n (%)	577 (36.6%)	378 (47.7%)	280 (52.3%)	<0.001	1235 (42.5%)
Aneuploid blastocysts, n (%)	806 (51.1%)	255 (32.1%)	167 (31.2%)	<0.001	1228 (42.3%)
Mosaic blastocysts, n (%)	195 (12.3%)	160 (20.2%)	88 (16.5%)	<0.001	440 (15.2%)
Blastocysts with *de novo* aneuploidy, n(%)	308 (19.5%)	255 (32.1%)	167 (31.2%)	<0.001	730 (25.1%)
Stimulation protocol					
PPOS	916 (58.0%)	427 (53.8%)	305 (57.0%)	–	1648 (56.7%)
GnRH agonist	455 (28.8%)	272 (34.3%)	146 (27.3%)	–	873 (30.0%)
GnRH antagonist	207 (13.2%)	94 (11.9%)	84 (15.7%)	–	385 (13.3%)
Sperm concentration (10^∧^6/ml)					
≥ 15	1383 (87.6%)	746 (94.1%)	454 (84.9%)	–	2583 (88.9%)
< 15	195 (12.4%)	47 (5.9%)	81 (15.1%)	–	323 (11.1%)
Sperm motility (PR + NP) (%)					
≥ 40	805 (51.0%)	448 (56.5%)	349 (65.2%)	–	1602 (55.1%)
< 40	773 (49.0%)	345 (43.5%)	186 (34.8%)	–	1304 (44.9%)
Sperm normal morphology (%)					
≥ 4	1269 (80.4%)	711 (89.7%)	455 (85.0%)	–	2435 (83.8%)
< 4	309 (19.6%)	82 (10.3%)	80 (15.0%)	–	471 (16.2%)
DFI (%)					
≤ 15	1126(71.4%)	502(63.3%)	280(52.3%)	–	1908(65.7%)
> 15	452(28.6%)	291(36.7%)	255(47.7%)	–	998(34.3%)
Blastocyst expansion, n (%)					
3	109 (6.9%)	49 (6.2%)	11 (2.1%)	–	169 (5.8%)
4	1289 (81.7%)	649 (81.8%)	468 (87.5%)	–	2406 (82.8%)
5	161 (10.2%)	79 (10.0%)	48 (9.0%)	–	288 (9.9%)
6	19 (1.2%)	16 (2.0%)	8 (1.5%)	–	43 (1.5%)
ICM grade, n (%)					
A	127 (8.0%)	64 (8.1%)	37 (6.9%)	–	228 (7.8%)
B	1178 (74.7%)	610 (76.9%)	401 (75.0%)	–	2189 (75.3%)
C	273 (17.3%)	119 (15.0%)	97 (18.1%)	–	489 (16.8%)
TE grade, n (%)					
A	76 (4.8%)	42 (5.3%)	14 (2.6%)	–	132 (4.5%)
B	1025 (65.0%)	497 (62.7%)	349 (65.2%)	–	1871 (64.4%)
C	477 (30.2%)	254 (32.0%)	172 (32.2%)	–	903 (31.1%)

Continuous variables are expressed as mean ± standard deviation (SD) or median (Q1, Q3). Categorical values are presented as number (percentages).

PGT-SR, Preimplantation Genetic Testing for Structural Rearrangements; PGT-A, Preimplantation Genetic Testing for Aneuploidy; PGT-M, Preimplantation Genetic Testing for Monogenic.

BMI, body mass index; AMH, Anti-Müllerian hormone; MII, second metaphase; high-quality blastocysts, based on Gardner morphological grading system with a grade of ≥ 3BB (including grades 3, 4, 5, 6, AA, AB, BA, and BB); DFI, DNA fragmentation index; ICM, inner cell mass; TE, trophectoderm.

### Univariate analysis of influencing factors for *de novo* aneuploidy

The percentages of *de novo* aneuploidy were summarized in terms of maternal parameters, semen parameters, and embryonic morphological parameters in **Table 2**. These factors included, in particular: maternal age, paternal age, AMH, maternal BMI, PGT indication (except for PGT-M group), COS protocol, sperm concentration, motility, morphology, DFI, degree of expansion, ICM grade, and TE grade. The statistical results showed that maternal age, paternal age, and expansion degree were significantly associated with *de novo* aneuploidy in the PGT-SR cohort (*P* < 0.001, *P* = 0.026 and *P* = 0.020). Although there was no statistically significant difference, we found that ICM grade and TE grade had a negative correlation with *de novo* aneuploidy. Within the PGT-A cohort, there were statistically significant associate in such abnormalities with maternal age, AMH, PGT indication, COS protocol, ICM grade and TE grade (*P* = 0.002, *P* = 0.002, *P* = 0.002, *P* = 0.003 and *P* = 0.034, respectively). Similarly, the rate of *de novo* aneuploidy was found to have a negative correlation with degree of blastocyst expansion, TE grade and ICM grade in the PGT-M cohort, however, the difference did not reach statistical significance (*P* > 0.05). It was noteworthy that there remained a positive correlation between maternal age and *de novo* aneuploidy; however, in the PGT-M cohort, this correlation was not statistically significant (adjusted *P* = 0.532).

**Table 2:** The univariate analysis of influencing factors for *de novo* aneuploidy.

Variables	PGT-SR	PGT-A	PGT-M
Maternal age			
< 30	17.6% (134/761)	15.8% (19/120)	28.6% (52/182)
30-34	18.1% (117/646)	22.7% (80/352)	29.6% (71/240)
≥ 35	48.7% (57/171)	48.6% (156/321)	38.9% (44/113)
*P* value	<0.001	0.002	0.532
Paternal age			
< 30	16.6% (93/599)	33.3% (27/81)	33.1% (40/121)
30-34	19.3% (142/737)	30.0% (106/353)	27.4% (69/252)
≥ 35	25.9% (73/282)	34.0% (122/359)	35.8% (58/162)
*P* value	0.026	0.556	0.519
AMH (ng/ml)			
< 1.1	24.3% (33/136)	65.1% (28/43)	41.4% (12/29)
1.1-3.5	18.7% (125/668)	37.3% (118/316)	32.9% (80/244)
> 3.5	19.4% (150/774)	25.1% (109/434)	28.6% (75/262)
*P* value	0.530	0.002	0.688
PGT indication ^&^			
A	19.6% (196/1002)	25.5% (129/505)	—
B	20.7% (87/421)	13.8% (9/65)	—
C	16.1% (25/155)	45.5% (106/223)	—
*P* value	0.570	0.002	—
Maternal BMI			
< 18.5	19.3% (22/114)	21.3% (10/47)	32% (8/25)
18.5-23.9	18.6% (201/1079)	32.3% (173/536)	30.2% (127/420)
24.0-27.9	21.3% (72/338)	32.4% (56/173)	33.3% (24/72)
≥ 28	27.7% (13/47)	43.2% (16/37)	44.4% (8/18)
*P* value	0.520	0.288	0.816
Stimulation protocol			
PPOS	20.3% (186/916)	36.8% (157/427)	33.4 (102/305)
GnRH agonist	20.2% (92/455)	26.1% (71/272)	28.1% (41/146)
GnRH antagonist	14.5% (30/207)	28.7% (27/94)	28.6% (24/84)
*P* value	0.380	0.026	0.749
Sperm concentration (10^∧^6/ml)			
≥ 15	20.0% (276/1383)	32.2% (240/746)	30.8% (140/454)
< 15	16.4% (32/195)	31.9% (15/47)	33.3% (27/81)
*P* value	0.450	0.971	0.786
Sperm motility (PR + NP) (%)			
≥ 40	18.8% (151/805)	29.2% (131/448)	31.2% (109/349)
< 40	20.3% (157/773)	35.9% (124/345)	31.2% (58/186)
*P* value	0.570	0.083	0.991
Sperm normal morphology (%)			
≥ 4	19.8% (251/1269)	32.6% (232/711)	31.0% (141/455)
< 4	18.4% (57/309)	28.0% (23/82)	32.5% (26/80)
*P* value	0.600	0.472	0.859
DFI (%)			
≤ 15	18.3% (206/1126)	30.9% (155/502)	30.0% (84/280)
> 15	22.6% (102/452)	34.4% (100/291)	32.5% (83/255)
*P* value	0.170	0.404	0.788
Expansion degree			
3	30.3% (33/109)	44.9% (22/49)	36.4% (4/11)
4	19.6% (252/1289)	32.0% (208/649)	33.1% (155/468)
5	13.7% (22/161)	26.6% (21/79)	14.6% (7/48)
6	5.3% (1/19)	25.0% (4/16)	12.5% (1/8)
*P* value	0.020	0.261	0.160
ICM grade			
A	16.5% (21/127)	15.6% (10/64)	21.6% (8/37)
B	19.5% (230/1178)	32.0% (195/610)	31.4% (126/401)
C	20.9% (57/273)	42.0% (50/119)	34.0% (33/97)
*P* value	0.640	0.003	0.754
TE grade			
A	14.5% (11/76)	16.7% (7/42)	7.1% (1/14)
B	18.8% (193/1025)	30.8% (153/497)	26.9% (94/349)
C	21.8% (104/477)	37.4% (95/254)	41.9% (72/172)
*P* value	0.460	0.034	0.006

Values are presented as number, n (%).

& In the PGT-SR cohort, a=Reciprocal translocation, b=Robertsonian translocation, c=inversion; in the PGT-A cohort, a=recurrent pregnancy loss, b=recurrent implantation failure, c=advanced maternal age; in the PGT-M cohort, indications were not subdivided.

*P < 0.05 was considered statistically significant, all *P* values in the univariate analysis using the Benjamini-Hochberg false discovery rate (FDR) method to control Type I error inflation.

### Multivariate analysis of influencing factors for *de novo* aneuploidy

**Figure 4:**
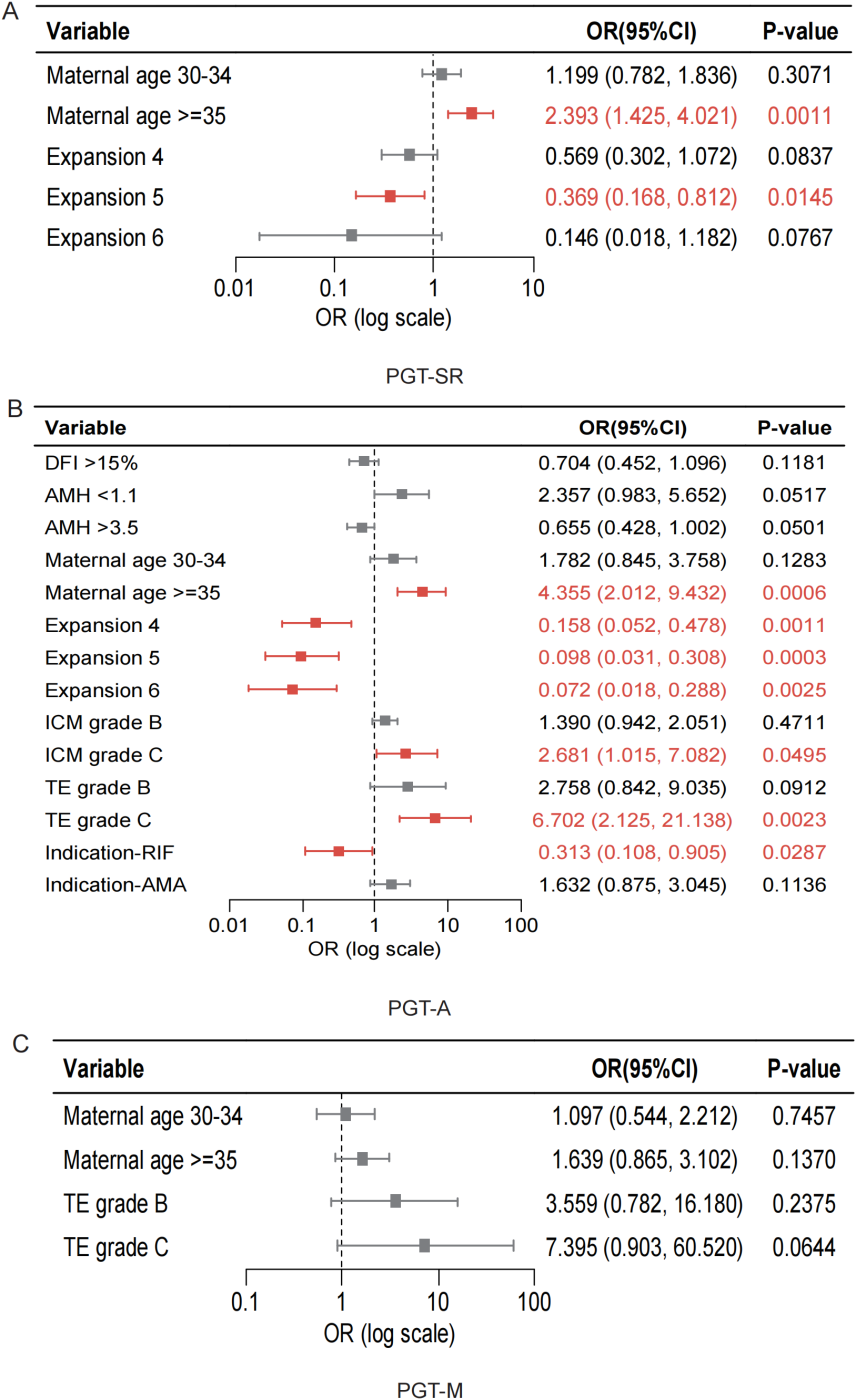
Multivariate forest plot employing the AIC-selected parsimonious model within the framework of the Generalized Linear Mixed Model (GLMM). **(A)** PGT-SR cohort. **(B)** PGT-A cohort. **(C)** PGT-M cohort. AMA, advanced maternal age; RIF, recurrent implantation failure.

Two multivariate GLMM models were developed. The clinically guided full model included those with *P* < 0.05 significance in any of the PGT cohort of univariate analysis or pre-specified variables of clinical interest. Due to the issue of multicollinearity, neither sperm concentration nor sperm morphology parameters were included in the full model. The clinically guided full model incorporated all factors, including maternal age, paternal age, AMH, maternal BMI, PGT indication (except for the PGT-M cohort), COS, sperm motility, sperm DFI, degree of expansion, ICM grade, and TE grade (**Table 3**). Backward stepwise selection based on AIC resulted in a parsimonious model with improved fit and fewer predictors. Owing to the heterogeneity of the three PGT datasets, the ultimate factors incorporated into the parsimonious model selected by the AIC differed among diverse PGT cohorts. The outcomes of the GLMM parsimonious model were presented in the form of forest plot in **Figure 4**. Variables that remained significant in both models were considered robust independent factors.

**Table 3:** The multivariate analysis of influencing factors for *de novo* aneuploidy.

Variables	PGT-SR		PGT-A		PGT-M	
	*P* value	OR (95% CI)	*P* value	OR (95% CI)	*P* value	OR (95% CI)
Maternal age						
< 30	Ref	—	Ref	—	Ref	—
30-34	0.570	1.120 (0.758-1.655)	0.198	1.925 (0.882-4.202)	0.799	1.160 (0.598-2.251)
≥ 35	0.030	2.027 (1.068-3.847)	0.008	5.262 (1.988-13.927)	0.904	1.175 (0.442-3.123)
Male age						
<30	Ref	—	Ref	—	Ref	—
30-34	0.607	1.150 (0.762-1.736)	0.724	0.825 (0.378-1.802)	0.904	0.873 (0.434-1.758)
≥35	0.607	1.212 (0.669-2.197)	0.704	0.783 (0.328-1.871)	0.884	1.486 (0.565-3.910)
AMH						
1.1-3.5	Ref	—	Ref	—	Ref	—
<1.1	0.775	0.894 (0.475-1.683)	0.152	2.356 (0.966-5.744)	0.861	1.299 (0.485-3.475)
>3.5	0.962	1.008 (0.714-1.425)	0.111	0.609 (0.386-0.963)	0.861	0.871 (0.508-1.493)
PGT indication ^&^						
A	Ref	—	Ref	—	—	—
B	0.853	1.044 (0.710-1.536)	0.043	0.291 (0.100-0.850)	—	—
C	0.638	0.827 (0.423-1.615)	0.198	1.671 (0.873-3.199)	—	—
Maternal BMI						
18.5-23.9	Ref	—	Ref	—	Ref	—
<18.5	0.760	0.866 (0.436-1.717)	0.952	0.958 (0.341-2.693)	0.799	1.520 (0.505-4.576)
24.0-27.9	0.539	1.190 (0.791-1.791)	0.198	0.641 (0.376-1.092)	0.904	1.106 (0.561-2.178)
≥28	0.775	1.232 (0.406-3.740)	0.570	0.685 (0.272-1.725)	0.904	1.276 (0.299-5.442)
Stimulation protocol						
PPOS	Ref	—	Ref	—	Ref	—
GnRH agonist	0.757	1.093 (0.737-1.620)	0952	0.984 (0.585-1.657)	0.799	0.779 (0.433-1.402)
GnRH antagonist	0.340	0.707 (0.413-1.212)	0376	0.683 (0.359-1.299)	0.904	0.869 (0.415-1.822)
**Sperm motility**	0.654	1.097 (0.776-1.550)	0.789	1.084 (0.696-1.690)	0.920	1.067 (0.618-1.843)
**Sperm DFI**	0.607	1.136 (0.779-1.658)	0.198	0.684 (0.425-1.100)	0.799	1.257 (0.722-2.188))
Expansion degree						
3	Ref	—	Ref	—	Ref	—
4	0.110	0.590 (0.308-1.128)	0.008	0.151 (0.049-0.471)	0.964	1.035 (0.233-4.606)
5	0.028	0.406 (0.181-0.912)	0.004	0.085 (0.024-0.310)	0.799	0.456 (0.080-2.592)
6	0.083	0.151 (0.018-1.287)	0.009	0.061 (0.011-0.352)	0.904	0.633 (0.039-10.226)
ICM grade						
A	Ref	—	Ref	—	Ref	—
B	0.786	1.115 (0.561-2.214)	0.628	1.375 (0.554-3.411)	0.827	0.755 (0.288-1.982)
C	0.853	1.088 (0.506-2.339)	0.136	2.770 (1.011-7.590)	0.920	1.110 (0.388-3.176)
TE grade						
A	Ref	—	Ref	—	Ref	—
B	0.548	0.738 (0.346-1.570)	0.198	2.689 (0.812-8.908)	0.730	3.241 (0.388-27.048)
C	0.980	0.990 (0.448-2.186)	0.013	6.653 (1.909-23.180)	0.587	7.389 (0.870-62.732)

Values are presented as number, n (%).

& In the PGT-SR cohort: a=reciprocal translocation, b=robertsonian translocation, c=inversion; in the PGT-A cohort: a=recurrent pregnancy loss, b=recurrent implantation failure, c=advanced maternal age; PGT-M group, indications were not subdivided.

*P < 0.05 was considered statistically significant, all *P* values in the multivariate analysis using the Benjamini-Hochberg false discovery rate (FDR) method to control Type I error inflation.

OR, odds ratio; CI, confidence interval.

In the PGT-SR cohort of two GLMM models, a significant correlation was observed between maternal age and *de novo* aneuploidy. It was discovered that females aged ≥35 exhibited significantly higher *de novo* an euploidy compared to those <30 [Odds Ratios (95% CI) = 2.027 (1.068-3.847), *P* = 0.030 in the full model; Odds Ratios (95% CI) = 2.393 (1.425-4.021), *P* = 0.001 in the parsimonious model]. However, there was no significant difference compared to those maternal aged 30-34 (*P* > 0.05). As the degree of blastocyst expansion increases, the probability of *de novo* aneuploidy gradually decreases, showing a negative correlation. Specifically, significant differences were observed between blastocysts with an expansion degree of 5 and those with an expansion degree of 3 (*P* = 0.028 in the full model and *P* = 0.014 in the parsimonious model), whereas the differences for an expansion degree of 6 were at the critical threshold (*P* = 0.083 and *P* = 0.076, respectively). No significant differences were observed in other factors (all *P* > 0.05).

Multivariate analysis in the PGT-A cohort revealed significant differences in maternal age, PGT-A indication, expansion degree, ICM grade, and TE grade. Compared with the younger age group (< 30 years), the risk of *de novo* aneuploidy was elevated in the older female group (aged ≥ 35 years) [Odds Ratios (95% CI) = 5.262 (1.988-13.927), *P* = 0.008 in the full model; Odds Ratios (95% CI) = 4.355 (2.012-9.432), *P* < 0.001 in the parsimonious model]. Controlling for other confounding factors, recurrent implantation failure (RIF) was an independent protective factor compared with recurrent pregnancy loss (*P* = 0.043 and *P* = 0.028, respectively). Consistent with the univariate analysis results, in multivariate analysis, expansion degree, ICM grade, and TE grade remained independent predictors of *de novo* aneuploidy, with significant differences observed in both models. Although AMH exhibited significant disparities in univariate analysis, its *P* values in both GLMM models were within the critical range. To further explore the role of AMH in the PGT-A cohort, a stratified analysis was conducted according to maternal age (**Table 4**). The statistical findings indicated that among the cohort of maternal aged ≥35 years, the *de novo* aneuploidy rates decreased gradually in the groups with AMH <1.1, 1.1-3.5, and >3.5, and statistically significant differences were observed (*P* < 0.001).

**Table 4:** AMH stratified by maternal age in the PGT-A cohort.

Variable	AMH < 1.1	AMH = 1.1-3.5	AMH > 3.5	*P* value
Maternal age < 30	33.3% (1/3)	9.7% (4/41)	18.4% (14/76)	0.230
30 ≤ Maternal age ≤ 34	12.5% (1/8)	32.5% (41/126)	17.4% (38/218)	0.005
Maternal age ≥ 35	81.2% (26/32)	48.9% (73/149)	40.7% (57/140)	<0.001

Values are presented as number, n (%).

*P < 0.05 was considered statistically significant.

Multivariate analysis of the PGT-M cohort showed that although maternal age had a positive correlation trend with *de novo* aneuploidy, the difference was not statistically significant. This might be due to the small sample size of the PGT-M cohort, which led to insufficient statistical power. In the PGT-M cohort, there was a clinically relevant tendency between TE grade and *de novo* aneuploidy rates. Compared with TE grade A embryos, grade B embryos showed an increased odds ratio of 3.56 (95% CI: 0.78-16.18, *P* = 0.238 in the parsimonious model), and grade C embryos showed an even higher odds ratio of 7.40 (95% CI: 0.90-60.52, *P* = 0.064 in the parsimonious model). Although these associations did not reach the conventional threshold of statistical significance (*P* < 0.05), the progressive increase in ORs with worse TE grade suggested a potential clinically relevant trend. Subsequently, we analyzed the predictive value of TE grade for *de novo* aneuploidy separately by stratifying according to maternal age. Significant differences in *de novo* aneuploidy rates were observed across different TE grades in the maternal age groups of 30–34 years and ≥35 years (**Table 5**).

**Table 5:** TE grade stratified by maternal age in the PGT-M cohort.

Variable	TE = A	TE = B	TE = C	*P* value
Maternal age < 30	0% (0/5)	33.7% (33/118)	32.2%(19/59)	0.420
30 ≤ Maternal age ≤ 34	0% (0/7)	25.3% (40/158)	41.3% (31/75)	0.010
Maternal age ≥ 35	50% (1/2)	28.8% (21/73)	57.9% (22/38)	0.005

*The Fisher-Freeman-Halton exact test was employed, and *P* < 0.05 was regarded as statistically significant.

## Discussion

In this research, a comprehensive assessment was carried out on the factors that influence *de novo* aneuploidy in three parallel PGT cohorts. In order to account for within-cycle clustering of blastocysts, a multi-factor mixed-effects model (generalized linear mixed model GLMM) with cycle ID as the random intercept to analyze the data in multivariate analysis. Simultaneously, the use of both a clinically guided full GLMM model and an AIC-selected parsimonious GLMM model allowed us to balance clinical plausibility with statistical parsimony, enhancing the robustness of our conclusions. In order to regulate the false discovery rate (FDR) brought about by multiple comparisons in both univariate and multivariate analyses, all *P* values were adjusted via the Benjamini-Hochberg procedure.

In our study, advanced maternal age (≥ 35 years) emerged as a consistent risk factor across all three PGT cohorts, with odds ratios exceeding 1 in the PGT-SR, PGT-A, and PGT-M cohorts. This finding aligns with well-established reproductive biology principles: advanced maternal age is associated with increased rates of aneuploidy and adverse pregnancy outcomes (27). Notably, the association was statistically significant in both the PGT-SR and PGT-A cohorts, with the stronger effect observed in the PGT-A cohort (OR = 4.355, 95%CI: 2.012-9.432, *P* < 0.001 in the parsimonious model). This stronger effect in PGT-A cohort may be biologically plausible: PGT-A is primarily performed for patients at higher risk of embryonic aneuploidy (e.g. AMA, RIF), where the impact of advanced maternal age on oocyte quality and embryo aneuploidy is most pronounced. The significant association in the PGT-SR cohort further confirms that in balanced translocation or inversion carriers, maternal age remains an independently predictive factor for *de novo* aneuploidy. In contrast, while a trend toward increased risk was observed in the PGT-M cohort, the association did not reach statistical significance. The relatively smaller sample size in this cohort may have limited statistical power to detect a significant effect. The impact of advanced maternal age could be attributed to the heightened risk of aneuplodies in oocytes and embryos as maternal age advances, primarily resulting from recombination errors during early meiosis, malfunctioning spindle assembly checkpoints at Metaphase I, and the loss of centromere cohesion (28).

AMH is a well-established marker for ovarian reserve and found to correlate with several outcomes in reproductive medicine, most reliably oocyte yield during ART cycles (29). Previous studies have shown that AMH independently predicts aneuploidy, and there was a correlation between AMH levels and the rate of embryonic aneuploidy in PGT-A patients (30, 31). A retrospective study grouped PGT-A cycles into three groups by AMH levels: AMH < 1.08; 1.08 ≤ AMH < 3.34; and AMH ≥ 3.34. The results showed that the aneuploidy rate of embryos decreased gradually across these three groups (30). These were in line with the conclusion of our research in the PGT-A cohort. In the PGT-A cohort, AMH showed significant differences in univariate analysis, a borderline significant trend was observed in the multivariate model, which was further clarified by maternal age stratified analysis. Age-stratified analysis suggested that the association between AMH and *de novo* aneuploidy was more apparent among women aged ≥ 35 years. In women aged ≥ 35 years, there was a clear dose-response relationship between lower AMH levels and higher *de novo* aneuploidy: the incidence was 81.2% in the AMH < 1.1ng/mL group, 48.9% in the AMH 1.1-3.5 ng/mL group, and 40.7% in the AMH > 3.5 ng/mL group (*P* < 0.001). This gradient was not detected in younger females (< 35 years), suggesting that the negative influence of low AMH on the risk of embryonic aneuploidy is most prominent in older women of reproductive age. While these findings persist, further validation is necessary across more ART centers with a larger population undergoing PGT-A. These findings align with the concept that AMH reflects both quantitative and qualitative aspects of ovarian reserve. In older women, diminished AMH not only indicates a reduced oocyte pool but also correlates with accelerated follicular depletion and increased rates of oocyte aneuploidy. In younger women, the protective effect of oocyte quality may mitigate the impact of lower AMH, explaining the lack of significant association in the < 35 age group. Research have shown that lower AMH levels may indicate granulosa cell hypoplasia, which affects oocyte maturation and chromosome stability (32). This may be the intrinsic mechanism of the association between aneuploidy and AMH level. The lack of a significant association within the PGT-SR and PGT-M cohorts might be attributed to the heterogeneity disparity among the study populations. Collectively, our results suggest that AMH is not a universal predictor of embryonic *de novo* aneuploidy but rather a clinically relevant marker, particularly in women of advanced maternal age undergoing PGT-A.

Controlled ovarian stimulation (COS) plays a significant role in ART by generating sufficient oocytes and blastocysts. Several studies have investigated which COS protocol leads to a higher blastocyst euploidy rate, however, the research findings have been inconsistent (33–35). A study including PGT-A cycles with the use of GnRH agonist, GnRH antagonist, or PPOS protocols found that the PPOS protocol cycle resulted in significantly lower euploidy rates compared to those using the GnRH-a and GnRH-ant protocols (36). However, Marca’s research found that blastocysts and euploid blastocysts count per patient and the number of euploid embryos per injected oocyte were similar between the PPOS and GnRH-ant protocol (37). Our study revealed that significant differences in embryonic aneuploidy rates were observed among the three protocols, suggesting a potential association in the PGT-A cohort. However, after adjustment for key confounders (including maternal age, AMH, and embryonic morphological parameters) in the multivariate full model, the association between stimulation protocol and embryonic aneuploidy was no longer statistically significant. This indicates that while baseline differences existed across protocols, the effect of COS protocol was largely mediated by other clinical factors (e.g., maternal age, ovarian reserve) in the multivariate context. Consequently, COS protocol was not considered an independent predictor in our study.

The precise link between semen parameters, such as sperm concentration, motility, morphology, DFI levels, and embryonic aneuploidy remains unclear (16). Emerging researches point to a rising recognition of the significance of paternal influences, especially in younger women, where sperm motility could be linked to embryo ploidy (38, 39). Some studies have shown that sperm DFI levels are significantly higher in patients with severe dyszoospermia than in the general population, which leads to higher rates of blastocyst aneuploidy (40, 41). However, another study revealed no significant correlations between semen parameters and embryonic aneuploidy (42). Kong’s research indicated that an elevated sperm DFI, as measured by the SCSA, does not significantly affect the euploidy rate of viable blastocysts in couples with advanced maternal age (43). No significant associations were observed between semen parameters and *de novo* aneuploidy across all PGT cohorts. In univariate analyses, sperm concentration, motility, normal morphology, and DFI showed no statistically significant differences in outcome rates (all *P* > 0.05). In multivariate models, neither sperm motility nor DFI emerged as independent predictors. Furthermore, all semen-related parameters were excluded from the parsimonious model during backward stepwise variable selection based on the Akaike Information Criterion (AIC). This indicates that, after adjusting for maternal age, AMH, and embryonic morphological parameters, semen parameters did not contribute significantly to model fit or predictive performance. These findings suggest that semen parameters do not exert an independent effect. It is important to consider that while abnormal semen parameters have been associated with sperm aneuploidy, it does not necessarily translate to embryonic aneuploidy of paternal origin, as aneuploid sperm may not result in viable embryos. Since all embryos detected during the PGT cycle were fertilized through ICSI, which may effectively prevent the impact of aneuploid sperm on embryo development.

Embryo quality has conventionally been evaluated via morphological features. Studies have shown that the parameters of blastocyst morphology have a significant correlation with embryonic ploidy, implantation rate, clinical pregnancy rate, and live birth rate (21, 44, 45). Our study comprehensively evaluated the predictive value of blastocyst morphological parameters, including expansion degree, ICM grade, and TE grade, across three PGT cohorts. Consistent with previous studies, higher blastocyst expansion degree was independently associated with a lower rate of embryonic aneuploidy, particularly in the PGT-A and PGT-SR cohorts. This observation supports the concept that blastocoel expansion reflects developmental competence and is closely linked to chromosomal integrity (44).

Notably, TE grade demonstrated a stronger and more consistent association with *de novo* aneuploidy risk than ICM grade in all three PGT cohorts. In the PGT-A cohort, poor TE (grade C) remained an independent risk factor even after multivariate adjustment and FDR correction. In the PGT-M cohort, although TE grade did not reach statistical significance in the GLMM full model, age-stratified analysis revealed a significant dose-response relationship in women aged ≥ 35 years, with markedly higher rate of *de novo* aneuploidy in embryos with poor TE. By contrast, ICM grade showed a relatively weak and inconsistent association. Although ICM was associated with *de novo* aneuploidy in some univariate comparisons, it lost significance in multivariate models and was excluded during AIC-driven stepwise selection in all cohorts. These findings are consistent with prior research, which has demonstrated that the morphology of the TE more precisely reflects ploidy status and outperforms the ICM in predicting euploidy (44, 45). It is hypothesized that the following factors may account for this phenomenon: Firstly, ploidy is a characteristic of the entire embryo rather than a trait specific to the ICM. Additionally, the TE morphology is more sensitive to chromosomal abnormalities and externally observable, thus exhibiting superior predictive performance. Secondly, TE grade is more objective and reproducible due to the application of uniform criteria. In contrast, the ICM, being smaller in size and located deeper within the blastocyst, is associated with greater inter-observer variability and poorer consistency. Thirdly, as the source of biopsied cells in PGT, the TE morphology is directly correlated with the ploidy results of the embryo. Collectively, our results confirm that TE grade exhibits an association with *de novo* aneuploidy, particularly in the PGT-A cohort, whereas ICM grade predictive value is slightly weaker than TE grade. The age-dependent effect of TE grade further highlights the importance of morphological evaluation in older women, in whom the predictive performance of morphology is amplified. These findings support the integration of blastocyst morphology into the clinical decision-making procedure and provide supplementary information for embryo prioritization.

In summary, our study had several key advantages. First, single-center sample collection ensured consistency in treatment, lab procedures, and embryonic evaluation during the study. In this context, parallel analysis of factors influencing aneuploidy in three distinct PGT cohorts was done. To our knowledge, this is currently the first and the most comprehensive study examining the influence factors of *de novo* aneuploidy following three types of PGT cohorts. Second, a GLMM model was used to address clustering effects and confounding factors, validating the findings. Also, using both a clinically guided full GLMM model and an AIC-selected parsimonious GLMM model balanced clinical plausibility and statistical parsimony, strengthening our conclusions. However, the study had some limitations. No formal sample size and power calculation was performed because this was a retrospective observational study. Subgroup analyses, particularly in the PGT-M cohort, may be underpowered to detect modest associations. Additionally, as trophectoderm cells evaluated morphologically were the same cells sampled for PGT biopsy, a methodological coupling effect might exist. There were also inherent limitations of retrospective cohort studies and selection bias from optimizing high quality blastocysts. In the PGT-SR group for couples with known chromosome rearrangements, only the *de novo* aneuploidy rate was used as the outcome measure. It’s unknown how intrinsic chromosomal abnormality affects gametes and embryos. Therefore, larger prospective, multicenter, and randomized controlled trials ought to be conducted to confirm our findings.

## Conclusions

In the present study, we comprehensively evaluated the predictive factors for *de novo* aneuploidy among three distinct PGT cohorts. Advanced maternal age (≥35 years) was identified as a consistent and robust risk factor across three PGT cohorts, with a particularly strong effect in the PGT-A cohort. Blastocyst expansion degree was an independent predictor in the PGT-A and PGT-SR cohorts, while TE grade and ICM grade only exhibited significant associations in the PGT-A cohort. In the PGT-M cohort, TE grade exhibited a suggestive association that became statistically significant in women aged ≥ 35 years, indicating an age-dependent effect. AMH was not an independent predictor overall but was associated with aneuploidy in older women within the PGT-A cohort. By contrast, paternal age, maternal BMI, semen parameters, and stimulation protocols showed no independent predictive value after multivariate adjustment and AIC-based stepwise selection. These findings highlight the predominant roles of maternal age and embryo morphology in determining PGT outcomes, with distinct predictive patterns across different PGT cohorts. This will facilitate the provision of genetic counseling and embryo selection for PGT populations.

## Data Availability

The original contributions presented in the study are included in the article/supplementary material. Further inquiries can be directed to the corresponding authors.
